# Comparison of intestinal and pharyngeal microbiota in preterm infants on the first day of life and the characteristics of pharyngeal microbiota in infants delivered by cesarean section or vaginally

**DOI:** 10.3389/fped.2024.1411887

**Published:** 2024-10-08

**Authors:** Jing He, Lijuan Wang, Ying Ruan, Xinyan Yan, Qingju Liu, Boren Chen, Sen Yang, Lijun Du

**Affiliations:** Department of Pediatrics, Chengdu Fifth People’s Hospital, Chengdu, China

**Keywords:** late preterm infants, intestinal microbiota, pharyngeal microbiota, mode of delivery, sequencing 16S rRNA, OTU, phylum and genus level, α and β diversity

## Abstract

**Background:**

This study aimed to explore the distribution of intestinal and pharyngeal microbiota on the first day of life in preterm infants and compare the composition of microbiota in infants delivered by cesarean section or vaginally.

**Methods:**

This study included 44 late preterm infants with a gestational age of 34–36 ^+ 6^ weeks. Stool and throat swab samples were collected from the preterm infants on the first day of life. The infants were divided into cesarean section and vaginal delivery groups. Illumina NovaSeq high-throughput sequencing technology was used to sequence the V3-V4 hypervariable region of the 16S rRNA gene of all bacteria in the samples. Venn diagram was used to identify shared operational taxonomic units (OTUs) in the intestines and pharynges. Microbial analysis was conducted at the phylum and genus levels, and α and *β* diversity comparisons were performed.

**Results:**

(1) Gestational age may have significantly affected the microbial colonization of the intestines and pharynges of preterm infants on the first day after birth (*p* ≤ 0.001). (2) More OTUs were detected in the pharynx than in the intestines, both have a total of 819 shared OTUs. *Proteobacteria*, *Firmicutes*, and *Bacteroidota* were the dominant phyla in both. At the genus level, Streptococcus had a lower relative abundance in stool samples (0.5%) compared to throat samples (0.5% vs. 22.2%, *p* = 0.003). 3) The relative abundance of *Streptococcus* in pharyngeal samples was 26.2% in the cesarean section group much higher than the 3.8% in the vaginal delivery group (*p* = 0.01).

**Conclusion:**

The early postnatal period is a critical time for the establishment of an infant's microbiota. Gestational age at birth may influence microbial colonization, while birth weight, gender, and mode of delivery do not. The intestinal and pharyngeal microbiota composition of preterm infants on the first day after birth showed high similarity, but larger samples are needed for further validation.

## Introduction

The human body contains millions of microorganisms, which gradually establish themselves in the first 1 to 3 years after birth and remain relatively stable throughout one's life (1, 2). These microorganisms inhabit various parts of the body, with the gastrointestinal tract being the largest microbial colonization (3). The intestinal microbiota plays crucial roles in human health by stimulating the development and maturation of the immune system, promoting intestinal homeostasis, resisting pathogens, aiding the digestion process of dietary fiber, and facilitating nutrient absorption. They are closely linked to the occurrence and development of various diseases and potentially have a role in predicting disease risks or providing new approaches for disease treatment (4). Numerous studies have indicated that the intestinal microbiota is involved in the pathogenesis of various childhood diseases, including necrotizing enterocolitis (NEC) in newborns (5), childhood asthma, allergic diseases (such as atopic dermatitis, allergic rhinitis, eczema), type 1 diabetes (6), obesity (7), and chronic immune-mediated inflammatory diseases (3). Moreover, the composition of the intestinal microbiota can influence the occurrence and development of these diseases in adulthood (3).

The pharynx is a part of the upper respiratory tract, a shared passage for the respiratory and digestive systems, and is exposed to various external and internal microorganisms (8). The composition of the pharyngeal microbiota is not only associated with oral health (9) and cardiovascular diseases (10) but has also been linked to the diagnosis of allergies, asthma (11), weight gain (12), and autism spectrum disorders (13). Therefore, the pharynx serves as a collection point for potentially pathogenic microorganisms, which may cause local inflammation and even lead to lung diseases (14). Studies by Man et al. have shown that during lower respiratory tract infections, nasopharyngeal samples can serve as representatives of the lung microbiota (15). Furthermore, the composition of the pharyngeal microbiota is related to susceptibility to upper or lower respiratory tract infections over time (16). Hence, the pharyngeal microbiota can reflect the characteristics of the entire respiratory tract microbiota to some extent, and thus throat swab samples can provide insights into the composition of the lung microbiota.

The establishment of a normal microbiota has a positive impact on immunity against pathogens (2). Collado et al. used next-generation sequencing technology to detect bacterial sequences in amniotic fluid from 15 healthy full-term pregnancies, indicating the presence of microbial communities in human amniotic fluid. The detection of microbial populations in healthy full-term amniotic fluid indicates that newborn colonization and the impact of microorganisms on infant health and development have already begun before birth (17). During fetal development, the intestinal microbiota is primarily composed of low-density bacteria such as *Streptococcus, Enterococcus, Lactobacillus*, and *Staphylococcus*. These may have originated from the mother's GI tract or amniotic fluid (18). During birth, the infant comes into contact with the maternal vaginal and intestinal microbiota, leading to the rapid colonization of facultative anaerobic bacteria like *Streptococcus, Enterobacteria, Enterococcus,* and *Staphylococcus (*19). As the colonization process continues, the abundance of obligate anaerobic bacteria such as *Bacteroides, Clostridium*, and *Bifidobacterium* increases, gradually replacing facultative anaerobic bacteria (20). Perrone et al. (21) collected urine samples from pregnant women three days before delivery and from healthy full-term infants within 48 h of birth. They utilized proton nuclear magnetic resonance (1H NMR) spectroscopy and evaluated the NMR urine spectra through Principal Component Analysis. The study found a significant correlation between the urinary metabolic profiles of newborns and their mothers. This suggests that newborns are likely programmed by maternal metabolism, and this programming may be influenced as early as the commencement of pregnancy. Additionally, Vélez-Ixta et al. (22) collected feces from Mexican pregnant women and their infants, as well as breast milk, for microbial and metabolomic analysis, and found similarities between the infant gut microbiota and breast milk components. A shared bacterial community exists within the mother-infant dyad, suggesting that the infant's microbial community may originate from vertical transmission through breast milk and that maternal-infant interactions during breastfeeding could impact the infant throughout its life. However, some researchers have found negligible bacterial biomass in amniotic fluid by testing 24 amniotic fluids from healthy full-term pregnancies (23). In any case, bacteria and/or viruses may already be colonized early in pregnancy. In contrast, preterm labor is associated with alterations in the bacterial microbiota of the placenta and amniotic fluid (24, 25).

The mode of delivery significantly impacts the composition of early-life intestinal microbiota (26). Dominguez-Bello MG et al. showed that infants delivered vaginally acquired a bacterial community similar to their own mother's vaginal microbiota, consisting of *Lactobacillus, Prevotella,* or *Sneathia spp*., and that infants delivered by cesarean section carried a bacterial community similar to that found on the surface of the skin, with *Staphylococcus, Corynebacterium*, and *Propionibacterium spp*. as the predominant organisms (27). Cesarean section delays microbial colonization (28). Furthermore, the intestinal microbiota of cesarean-born infants is more diverse but contains a higher number of potentially pathogenic bacteria, such as *hemolytic staphylococcus* and *Haemophilus parainfluenza*. In contrast, the intestinal microbiota of vaginally delivered infants is dominated by beneficial bacteria like *Bifidobacterium* (29, 30).

Infancy is a critical period for establishing microbiota-host interactions. This interaction may persist into adulthood and influence the host's health (31). Gestational age at birth also affects early-life microbial colonization. Premature infants often have immature immune systems, they may suffer from lack or delayed oral feeding, and exposure to broad-spectrum antibiotics. These disrupt the establishment of their intestinal microbiota (32). Compared to healthy full-term infants, premature infants have lower diversity and greater individual variation in their intestinal microbiota (33). Intestinal microbial colonization in premature infants is also slower, with an increase in potential pathogenic bacteria (especially *Escherichia coli*). In the meantime, there is delayed colonization by beneficial bacteria like *Bifidobacterium* and *Lactobacillus* (34).

In this study, Illumina NovaSeq high-throughput sequencing technology was employed, focusing on late preterm infants born between 34 and 36 ^+ 6^ weeks of gestation. Stool and throat swab samples were collected within the first day after birth to analyze the characteristics of the intestinal and pharyngeal microbiota, investigate their similarities, and explore the differences in composition and characteristics in infants delivered by cesarean section or vaginally. This research aimed to provide preliminary data on the role of intestinal and pharyngeal microbiota in the growth and development of premature infants.

## Material and methods

### Study recruitment and sample collection

The samples used in this study were all obtained from premature infants hospitalized in the NICU of Chengdu Fifth People's Hospital. The selection criteria were as follows: (1) Gestational age between 34 and 36 ^+ 6^ weeks. (2) Admission to the neonatology department for treatment immediately after birth. The exclusion criteria were as follows: (1) Congenital malformations or congenital genetic metabolic diseases. (2) Hypoxic-ischemic encephalopathy (HIE), severe asphyxia, immunodeficiency, or severe infections. (3) Gastrointestinal conditions such as perforation, etc. (4) Maternal use of antibiotics for more than 1 day before delivery. (5) Use of probiotics or prebiotics by the mother or the neonate during the perinatal period. (6) Lack of consent from the guardian to participate or the guardian to withdraw from the study.

Between April 2022 and September 2022, a total of 44 premature infants who met the inclusion criteria were included in the study. Within 30 min after birth, sterile throat swabs were used to collect pharyngeal secretions. Within 24 h after birth, when preterm infants passed their first bowel movement (the timing of the first bowel movement varies for each preterm infant), immediately collect 5–10 g in the center of a fresh stool using a sterile stool sample collector in a sterile cryopreservation tube, taking care to avoid mixing the feces and urine during collection. Following collection, the stool and throat swab samples were promptly placed in a freezer at −20°C and stored in a −80°C ultra-low-temperature freezer within 24 h for further analysis. In the process of collecting samples, we considered clinical covariates that may affect the research results (i.e., non-randomized factors such as age, birth weight, disease severity, etc.), and ensured that each sample had corresponding clinical covariate data to match them, thereby providing more accurate research results. This research protocol was approved by the ethics committee of Chengdu Fifth People's Hospital, and all participating families signed written informed consent forms.

### DNA extraction and amplification

Total genome DNA from the samples was extracted using the cetyltrimethylammonium bromide [CTAB, (Nobleryder, China)] method. DNA concentration and purity were monitored on 1% agarose gels. According to the concentration, DNA was diluted to 1 ng/µl using sterile water. The diluted genomic DNA served as a template for PCR amplification (BIO-RAD, T100, USA). Specific primers targeting the V3-V4 hypervariable region of the 16SrDNA gene, namely 341F and 806R [Shenggong Bioengineering (Shanghai) Co., Ltd], were used for amplification. All DNA samples were amplified following this protocol (35). Use TruSeq ® DNA PCR Free Sample Preparation Kit (Illumina, San Diego, CA, USA) for library construction. The resulting libraries were sequenced using an Illumina NovaSeq6000 platform (Illumina, San Diego, CA, USA), and sequencing services were provided by Beijing Novogene Genomics Technology Co. Ltd in China.

### Data processing and bioinformatics analysis

Based on the barcode sequences and PCR amplification primer sequences (V3-F: CCTAYGGGRBGCASCAG; V4-R: GGACTACNNGGGTATCTAAT), the reads from each sample were assembled using the FLASH software (36) to obtain Raw Tags. Subsequently, the fastp software was employed to obtain high-quality Clean Tags. Finally, the Clean Tags were aligned against a database using the Vsearch software (37) to obtain the final valid data, known as Effective Tags. The Uparse software (Uparse v7.0.1001, http://www.drive5.com/uparse/) (36, 37) was used to cluster all Effective Tags from all samples. The sequences were clustered into Operational Taxonomic Units (OTUs) with a default threshold of 97% identity. Representative sequences were selected from the OTUs based on the algorithm's principles, choosing the most abundant sequence within each OTU as the representative sequence. The taxonomic annotation of OTU sequences was conducted using the Mothur method against the SILVA138 SSUrRNA database (http://www.arb-silva.de/) (38, 39) with a threshold of 0.8∼1. Taxonomic information was obtained at various taxonomic levels, and in this study, species abundance and structure were analyzed at the phylum and genus levels. QIIME2 software was used to calculate diversity indices such as Shannon and chao1, and inter-group differences in α (within-sample) and β (between-sample) diversity were analyzed. Unifrac distances were calculated, and R software was employed to generate dimensionality reduction plots, including PCoA (Principal Coordinates Analysis).

### Statistical analysis

Statistical analysis was conducted using SPSS version 26.0 software. Categorical data were presented as percentages. To identify significant differences between groups at various taxonomic levels, *T*-tests or Wilcoxon tests were used. Fisher's exact test was used for the analysis of categorical variables. A *p*-value of less than 0.05 was considered statistically significant.

## Results

### Clinical information of subjects

During the study period, a total of 44 premature infants who met the inclusion criteria were included in the study. All pharyngeal samples were collected within 30 min of birth, and all stool samples were collected within the first day of life from the first meconium. Clinical information about the infants was obtained from their medical records, including gestational age, birth weight, gender, mode of delivery, stool sample collection time, whether infants were fed before stool samples were collected, feeding styles, infants with or without antibiotics, Whether the infant use antibiotics before sample collection, cause of preterm labor, PROM time, neonatal resuscitation, Apgar scores, either invasive (intubation) or noninvasive support (CPAP), mother's age and pregnancy complications, Vaginal infection, and antenatal steroid treatment, and so on (Supplementary Schedule 1, Table 1). The mean gestational age was 35.4 ± 0.9 weeks, mean birth weight was 2,419.8 ± 409.7 g. Out of the 44 infants, 10 (22.7%) were of vaginal delivery, and 34 (77.3%) were of cesarean delivery. Among them, there were 17 (38.6%) female infants and 27 (61.4%) male infants (Table 1).

**Table 1:** General characteristics of the study subjects.

Characteristics	Value
GA, weeks, mean (SD)	35.4 ± 0.9
Birth weight (g), mean (SD)	2,419.8 ± 409.7
Gender, *n* (%)	
Male	17 (38.6%)
Female	27 (61.4%)
Delivery, *n* (%)	
Cesarean	34 (77.3%)
Vaginal	10 (22.7%)
Stool sample collection time after birth (hours), mean (SD)	6.4 ± 5.1
Apgar scores, mean (SD)	
1-min	9.2 ± 1.1
5-min	9.9 ± 0.25
Were infants fed before stool samples were collected? *n* (%)	
Yes	34 (77.3%)
No	10 (22.7%)
Whether the infant use antibiotics before sample collection? *n* (%)	
Yes	13 (29.5%)
No	31 (70.5%)
PROM (hours), *n* (%)	
Yes	15 (34.1%)
No	29 (65.9%)
Neonatal resuscitation, *n* (%)	
Yes	6 (13.6%)
No	38 (86.4%)
Either invasive (intubation) or noninvasive support (CPAP), *n* (%)	
Intubation	0
CPAP	11 (25%)
NO	33 (75%)
Mother's age (years), mean (SD)	29.2 ± 3.5
Antenatal steroid treatment, *n* (%)	
Yes	10 (22.7%)
No	34(77.3%)

GA, gestational age; PROM, Prom premature rupture of membranes.

### Bacterial detection in stool and throat

**Figure 1:**
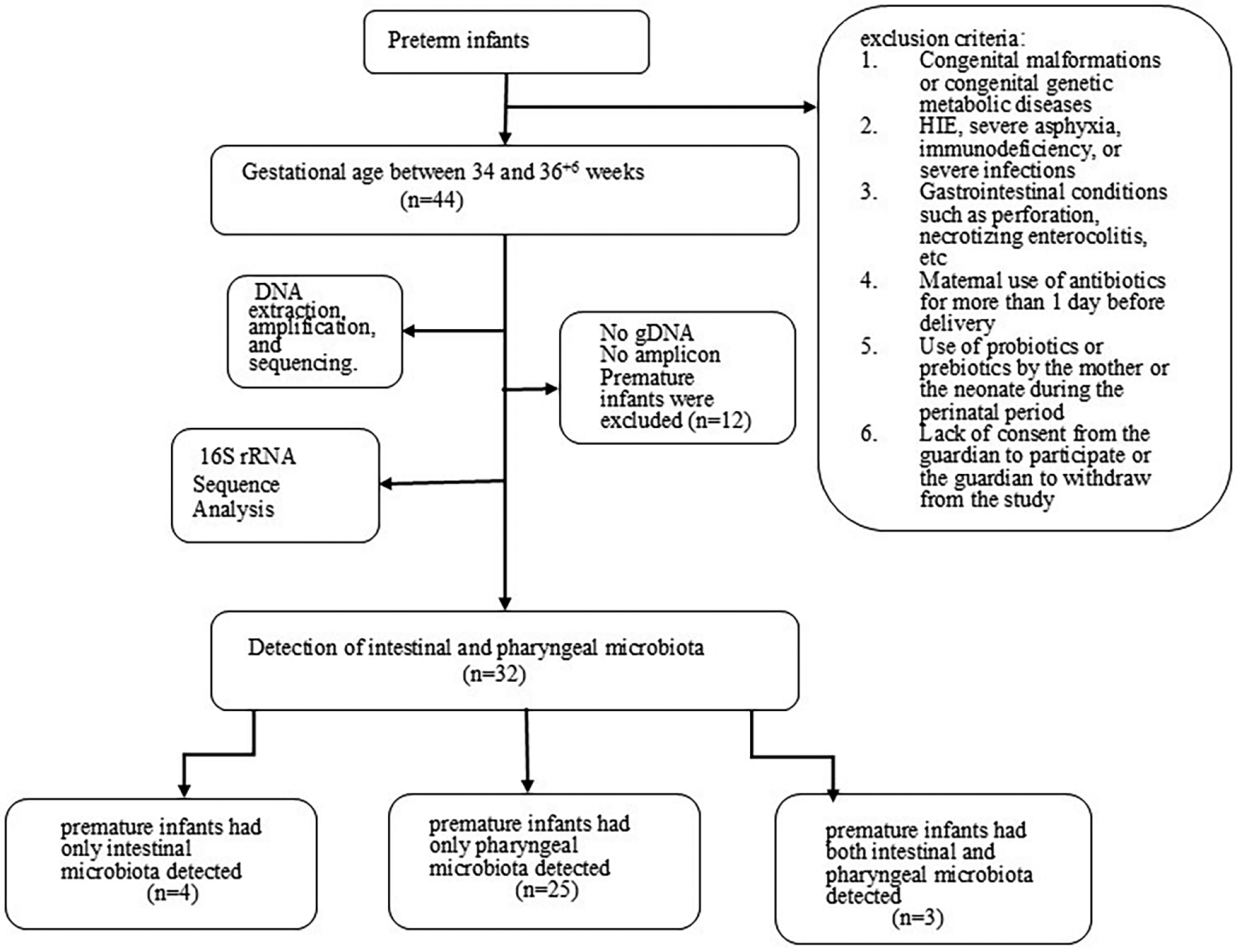
Flowchart of the study population.

A total of 44 stool samples and 44 pharyngeal samples were collected from preterm infants. After DNA extraction, amplification, and sequencing. The sequencing sequence is double-ended and has a length of 250 bp (The number of readings for each sample is shown in the Supplementary Materials), we were able to detect microbiota in 32 premature infants. Among them, 4 premature infants had only intestinal microbiota detected, 25 premature infants had only pharyngeal microbiota detected, and 3 premature infants had both intestinal and pharyngeal microbiota detected. The bacterial detection rate in stool samples was 7/44 (15.9%), while the detection rate in throat swab samples was 28/44 (63.6%). Twelve premature infants were excluded from the analysis due to either failed gDNA extraction or insufficient library amplification (Figure 1). Table 2 compares the clinical characteristics of preterm infants with and without detectable microbiota in pharyngeal and intestinal samples. Gestational age has some effect on microbial colonization (*p* ≤ 0.001).

**Table 2:** Demographic comparison of preterm infants with and without detectable microbiota in pharyngeal and intestinal samples.

Characteristics	Intestinal samples			Pharyngeal samples		
	Detected group	Not detected group	*p*	Detected group	Not detected group	*p*
Number	7	37	–	28	16	–
Maternal age (years)*	26–35 (29)	23–36 (29)	0.60	23–25 (29)	25–36 (31)	0.45
Delivery (Cesarean/Vaginal)	7/0	27/10	0.24	23/5	11/5	0.59
Sex of infant (male/Female)	5/2	12/25	0.15	15/13	2/14	0.43
Gestational age (weeks)*	34.3–36.1 (35.1)	34.0–36.6 (35.5)	0.001	34.0–36.6 (35.2)	34.2–36.6 (36.1)	<0.001
Birth weight of infant (g)*	2,310–3,060 (2,550)	1,510–3,270 (2,340)	0.310	1,510–3,120 (2,325)	1,810–3,270 (2,520)	0.315
Whether the infant use antibiotics before sample collection? No/Yes	5/2	26/11	0.19	22/6	9/7	0.50
Were infants fed before stool samples were collected? No/Yes	2/5	8/29	0.29	8/20	2/14	0.55
Stool sample collection time after birth (hours)*	0.5–12.0 (4.7)	0.5–20.0 (8.8)	0.06	0.5–18.0 (5.3)	0.5–20.0 (8.3)	0.06

* min - max value (mean) are provided in cells.

### Analysis of intestinal and pharyngeal microbiota

#### Composition of intestinal and pharyngeal microbiota at the phylum level

The intestinal microbiota was predominantly composed of the following bacterial phyla: *Proteobacteria* (57.4%), *Firmicutes* (36.1%), *Bacteroidota* (4.3%), *Actinobacteriota* (0.9%), and *Acidobacteriota* (0.2%). In contrast, the throat microbiota consisted mainly of *Firmicutes* (45.0%), *Proteobacteria* (37.9%), *Bacteroidota* (6.3%), *Actinobacteriota* (4.1%), and *Acidobacteriota* (0.5%).

**Figure 2:**
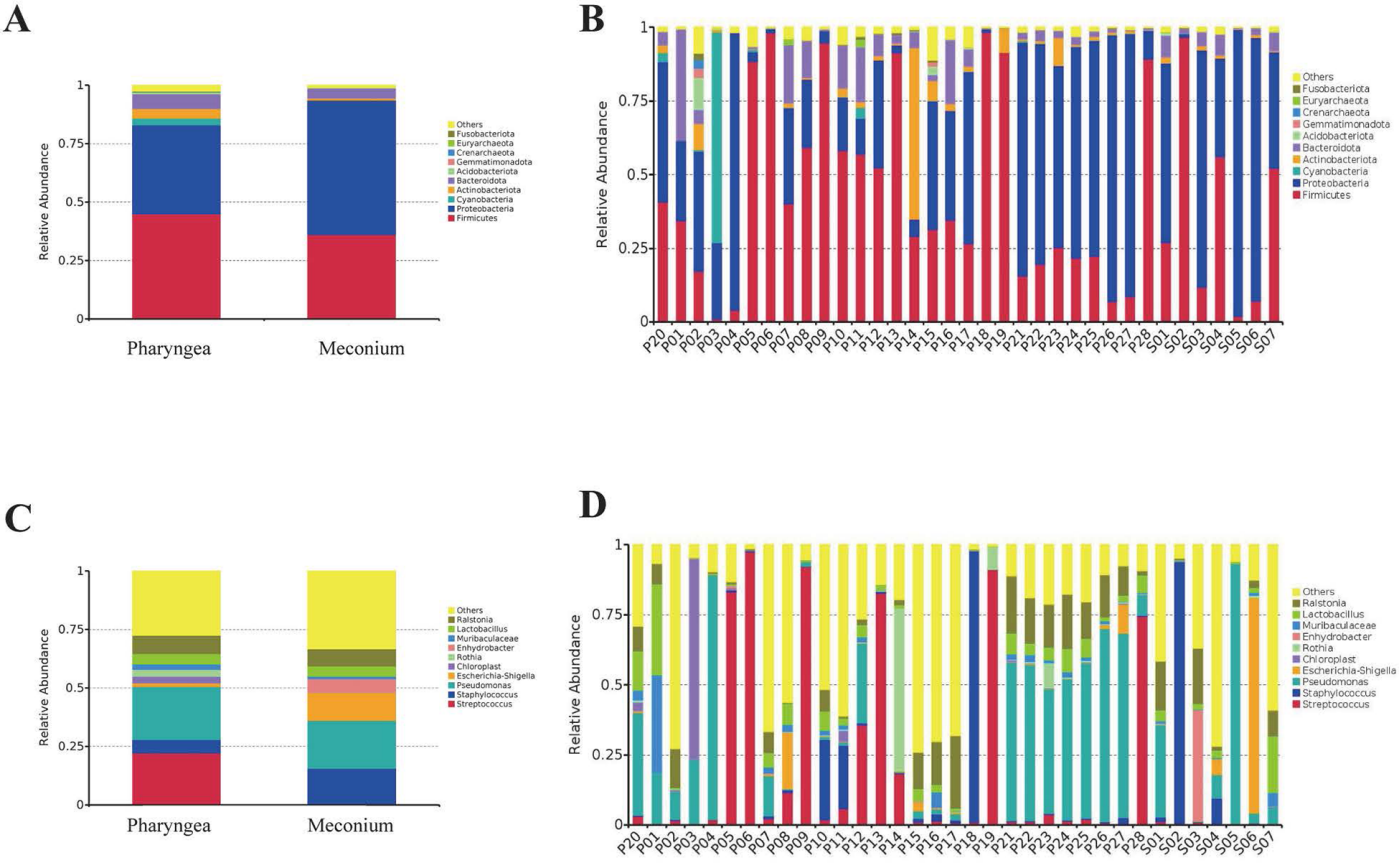
Composition of intestinal (*N* = 7) and pharyngeal (*N* = 28) microbiota at the phylum and genus level. **(A)** Relative abundance of intestinal and pharyngeal microbiota at the phylum level. **(B)** Composition of microbiota in each sample at the phylum level. **(C)** Relative abundance of intestinal and pharyngeal microbiota at the genus level. **(D)** Composition of microbiota in each sample at the genus level.

The relative abundance of *Proteobacteria* in stool and throat samples was 57.4% and 37.9%, respectively (*p* = 0.21). *Firmicutes* had a relative abundance of 36.1% in stool and 45.0% in throat samples (*p* = 0.55). *Bacteroidota* had a relative abundance of 4.3% in stool and 6.3% in throat samples (*p* = 0.33). *Actinobacteriota* had a relative abundance of 0.9% in stool and 4.1% in throat samples (*p* = 0.14). *Acidobacteriota* had a relative abundance of 0.2% in stool and 0.5% in throat samples (*p* = 0.39). These differences were not statistically significant (Figure 2A). The most abundant phyla in both intestinal and pharyngeal microbiota were *Proteobacteria* and *Firmicutes* (Figure 2B).

#### Composition of intestinal and pharyngeal microbiota at the genus level

The composition of the intestinal microbiota includes *Pseudomonas* (20.6%), *Staphylococcus* (15.0%), *Escherichia-Shigella* (11.9%), *Lactobacillus* (4.4%) and *Streptococcus* (0.5%). Pharyngeal microbiota composed of *Pseudomonas* (22.7%), *Streptococcus* (22.2%), *Staphylococcus* (5.7%), *Lactobacillus* (4.4%) and *Escherichia-Shigella* (1.5%) and others.

The relative abundance of *Pseudomonas* in stool and throat samples was 20.6% and 22.7%, respectively (*p* = 0.88). *Escherichia-Shigella* had a relative abundance of 11.9% in stool and 1.5% in throat samples (*p* = 0.38). *Staphylococcus* had a relative abundance of 15.0% in stool and 5.7% in throat samples (*p* = 0.52). *Lactobacillus* had a relative abundance of 4.4% in both stool and throat samples (*p* = 0.99). There were no statistically significant differences between intestinal and pharyngeal microbiota. However, *Streptococcus* had a relative abundance of 0.5% in stool and 22.2% in throat samples (*p* = 0.003), indicating a statistically significant difference (Figure 2C). The most abundant genera in both intestinal and pharyngeal microbiota were *Pseudomonas* (Figure 2D).

### Comparisons between intestinal and pharyngeal microbiota

#### Comparisons between intestinal and pharyngeal OTU

**Figure 3:**
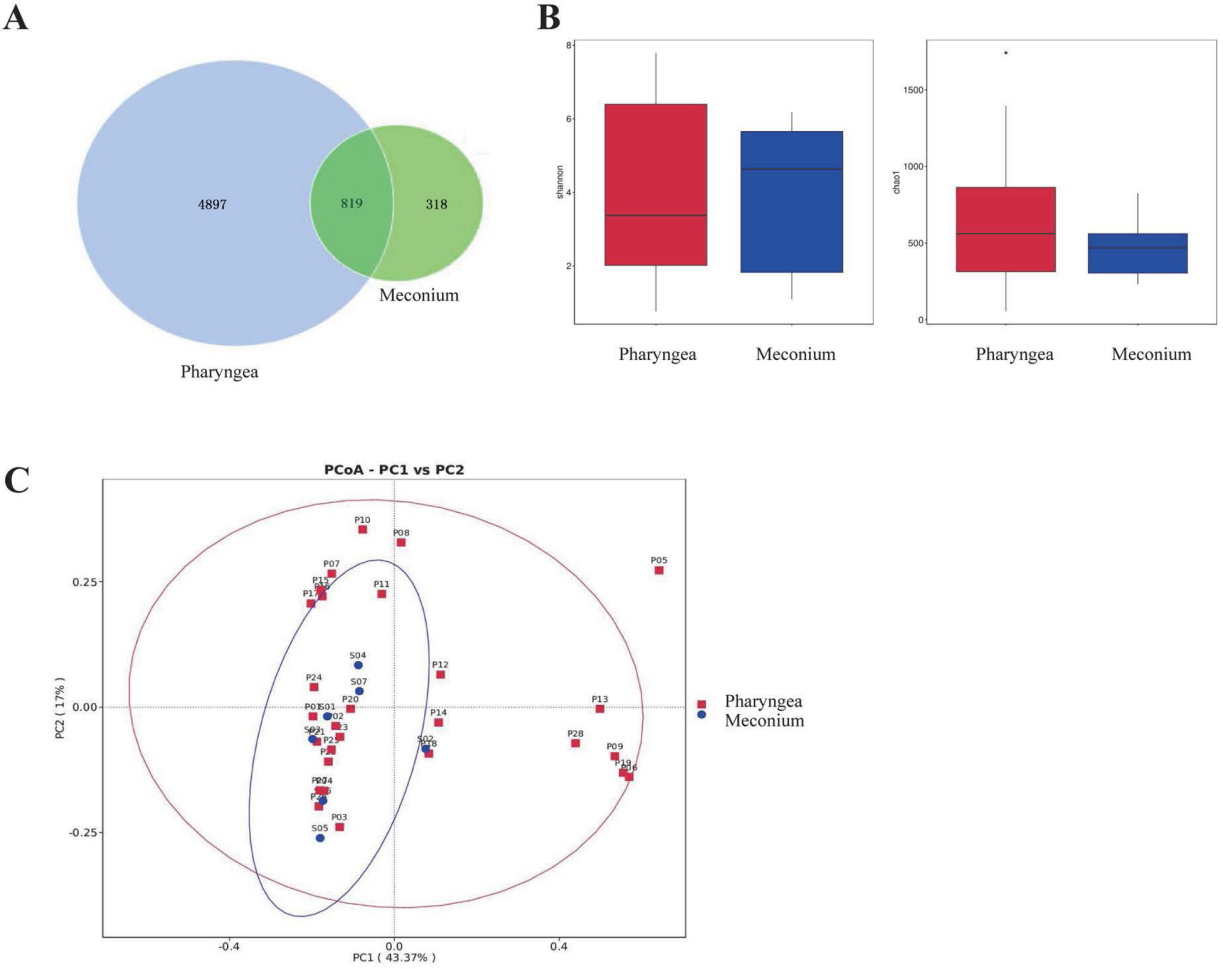
Comparison of intestinal (*N* = 7) and pharyngeal (*N* = 28) microbiota. **(A)** Intestinal and Pharyngeal OTUs. **(B)** Biodiversity comparison using Shannon and Chao1 index. **(C)** PCoA plot based on OTU abundance.

A total of 1,137 OTUs were detected in stool samples, while 5,716 OTUs were detected in throat samples. There were more OTUs detected in the throat than in the stool. However, both stool and throat samples shared 819 common OTUs (Figure 3A).

#### Alpha diversity comparison (Shannon index and Chao1 index)

To assess the differences in microbial diversity and abundance between the intestinal and pharyngeal microbiota, Shannon and Chao1 indices were used as estimators. The results showed that there were no statistically significant differences in the Shannon index (*p* = 0.93) or Chao1 index (*p* = 0.21) between the intestinal and pharyngeal microbiota of premature infants on the first day of life (Figure 3B).

#### Beta diversity comparison

To further compare the composition of the microbiota in the intestines and pharynges, beta diversity comparisons were conducted. PCoA was performed at the OTU level, where PC1 represents the first principal coordinate with a contribution rate of 43.37% to the overall microbial community. The vertical axis represents the second principal coordinate with a contribution rate of 17.00%. The results indicate that there is no significant difference in the composition of intestinal and pharyngeal microbiota in premature infants on the first day after birth (*p* > 0.99) (Figure 3C).

Each point represents the microbial community of the subjects’ stool or throat. The horizontal axis and vertical axis represent two selected principal component axes. The percentages represent the values of the differences in the principal components on the sample components. Each marker represents an individual sample, and points with different colors or shapes represent samples from different groups. The closer the points of the two samples are, the higher the similarity in species composition between the two samples.

### Comparison of pharyngeal microbiota composition in different delivery modes (cesarean section group vs. vaginal delivery group)

#### Comparison at the phylum level

At the phylum level, cesarean section infants had pharyngeal microbiota composed of *Firmicutes* (49.4%), *Proteobacteria* (36.1%), *Bacteroidota* (6.7%), *Actinobacteriota* (4.4%) and *Acidobacteriota* (0.2%). Infants born vaginally had pharyngeal microbiota composed of *Proteobacteria* (46.3%), *Firmicutes* (24.4%), *Bacteroidota* (4.5%), *Actinobacteriota* (2.6%), *Acidobacteriota* (2.2%), and others.

**Figure 4:**
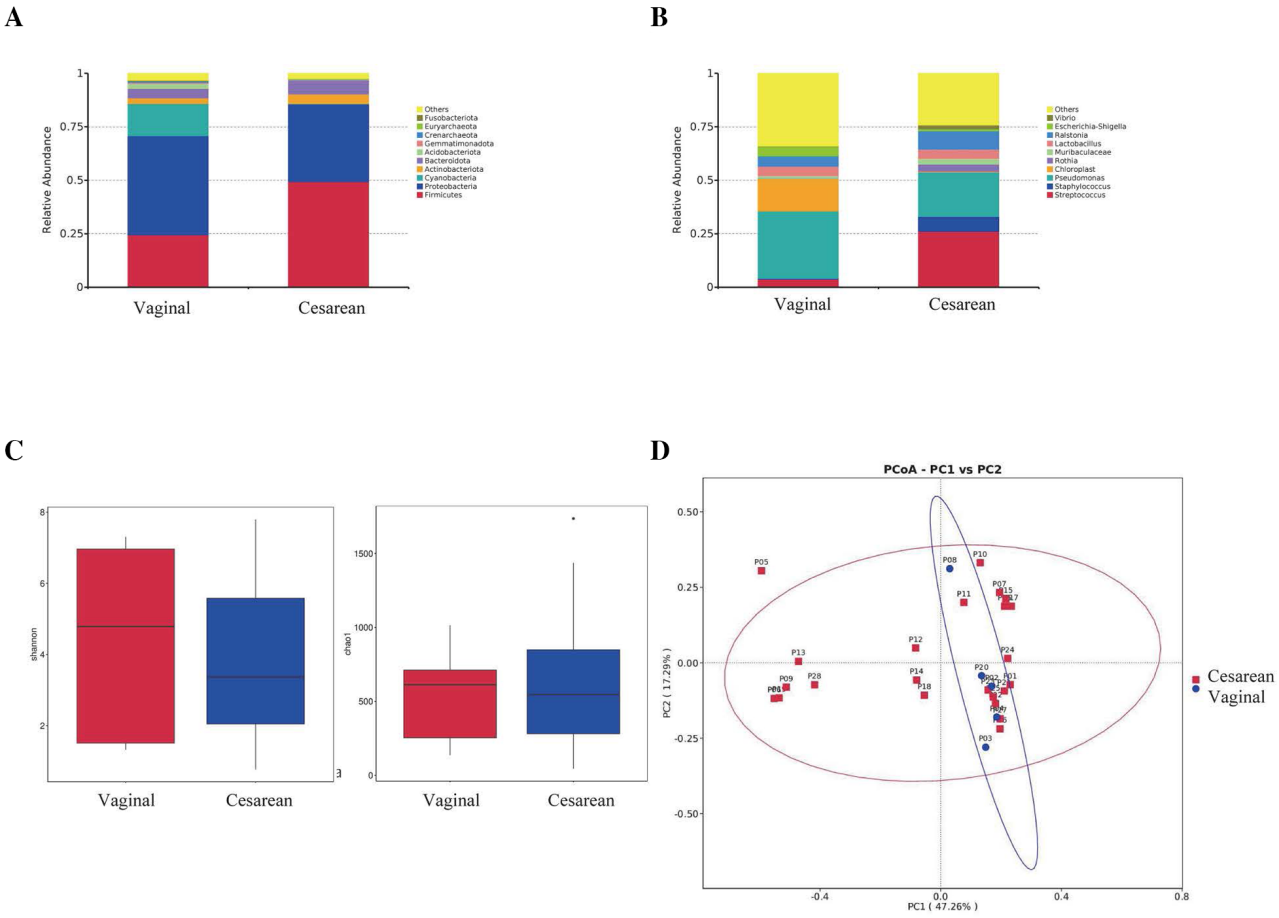
Comparison of pharyngeal microbiota between infants born via cesarian section (*N* = 27) and vaginal delivery (*N* = 5). **(A)** Comparison at the phylum level. **(B)** Comparison at the genus level. **(C)** Alpha diversity comparison using Shannon and Chao1 index. **(D)** PCoA plot based on OTU abundance.

*Proteobacteria* had a relative abundance of 36.1% in cesarean section births and 46.3% in vaginal deliveries (*p* = 0.51). *Firmicutes* had a relative abundance of 49.4% in cesarean section births and 24.4% in vaginal deliveries (*p* = 0.10). *Bacteroidota* had a relative abundance of 6.7% in cesarean section births and 4.5% in vaginal deliveries (*p* = 0.48). *Actinobacteriota* had a relative abundance of 4.4% in cesarean section births and 2.6% in vaginal deliveries (*p* = 0.53). *Acidobacteriota* had a relative abundance of 0.2% in cesarean section births and 2.2% in vaginal deliveries (*p* = 0.38). None of these differences were statistically significant (Figure 4A).

#### Comparison at the genus level

At the genus level, preterm infants born via cesarean section had pharyngeal microbiota mainly composed of *Pseudomonas* (20.8%), *Streptococcus* (26.2%), *Ralstonia* (8.6%), *Staphylococcus* (6.9%), *Lactobacillus* (4.4%), *Rothia* (3.4%), Muribaculaceae (2.5%), *Vibrio* (1.9%) and Escherichia-Shigella (0.9%). Infants born vaginally had pharyngeal microbiota mainly composed of *Pseudomonas* (31.6%), *Ralstonia* (4.8%), *Lactobacillus* (4.5%), Escherichia-Shigella (4.2%), *Streptococcus* (3.8%), *Muribaculaceae* (1.2%), *Vibrio* (0.4%), *Staphylococcus* (0.2%) and *Rothia* (0.2%).

*Pseudomonas* had a relative abundance of 20.8% in cesarean section births and 31.6% in vaginal deliveries (*p* = 0.53). *Staphylococcus* had a relative abundance of 6.9% in cesarean section births and 0.2% in vaginal deliveries (*p* = 0.14). *Escherichia-Shigella* had a relative abundance of 0.9% in cesarean section births and 4.2% in vaginal deliveries (*p* = 0.45). *Rothia* had a relative abundance of 3.4% in cesarean section births and 0.2% in vaginal deliveries (*p* = 0.22). *Muribaculaceae* had a relative abundance of 2.5% in cesarean section births and 1.2% in vaginal deliveries (*p* = 0.44). *Lactobacillus* had a relative abundance of 4.4% in cesarean section births and 4.5% in vaginal deliveries (*p* = 0.99). *Ralstonia* had a relative abundance of 8.6% in cesarean section births and 4.8% in vaginal deliveries (*p* = 0.29). *Vibrio* had a relative abundance of 1.9% in cesarean section births and 0.4% in vaginal deliveries (*p* = 0.06). None of these differences were statistically significant. On the other hand, *Streptococcus* had a relative abundance of 26.2% in cesarean section births and 3.8% in vaginal deliveries (*p* = 0.01), which was statistically significant (Figure 4B).

#### Alpha diversity comparison (Shannon index and Chao1 index)

Shannon and Chao1 indices were used as estimates to evaluate the diversity and abundance of the pharyngeal microbiota. Results showed no statistically significant differences in Shannon index (*p* = 0.70) or Chao1 index (*p* = 0.68) between the cesarean section and vaginal delivery groups (Figure 4C).

#### Beta diversity comparison

To further compare the composition of the pharyngeal microbiota between cesarean section and vaginal delivery groups, beta diversity was assessed. PCoA was performed at the OTU level, where PC1 represented 47.26% of the total microbiota variation, and the vertical axis represented the second principal coordinate, accounting for 17.29% of the variation. The results showed no significant differences in the composition of the pharyngeal microbiota between the cesarean section and vaginal delivery groups on the first day after birth (*p* = 0.25) (Figure 4D).

## Discussion

Microbial colonization is a complex and dynamic process. After birth, various factors such as changes in the external environment, feeding and diet, and antibiotic use can influence the composition of the microbiota. In this study, late preterm infants born between 34 and 36 ^+ 6^ weeks of gestation were chosen as the subjects. All pharyngeal samples were collected within 30 min of birth, and all stool samples were collected within the first day of life from the first meconium. Previous research has found that the mode of delivery can influence the colonization of the newborn's intestinal microbiota (40). In contrast, this study found that birth weight, and gender did not significantly influence the colonization of the intestinal and pharyngeal microbiota on the first day of life (*p* > 0.05). This may be related to the fact that our samples were collected immediately after birth when the colonization of environmental microbiota had not yet been completed. However, the relative abundance of *Streptococcus* in pharyngeal samples was 26.2% in the cesarean section group much higher than the 3.8% in the vaginal delivery group *(p* = 0.01). The study also found that gestational age at birth may influence microbiota colonization (*p* ≤ 0.001), which is consistent with the findings of Magne et al. (33). This study also found very low rates of detection of bacterial DNA within the first pass of meconium, which is similar to the results of previous studies (17). Previously published work suggests that meconium yields 0.2 ± 0.4 ng of prokaryotic DNA per mg of meconium, compared with 16.6 ± 6.4 ng of prokaryotic DNA per mg of stool at 1 year of age (41). Fetal feces have a tarry texture, are difficult to dissolve, and contain a high concentration of PCR inhibitors, making bacterial DNA extraction more difficult (42).

Generally, the microbiome of newborns, including both full-term and preterm infants, is primarily composed of four phyla: *Firmicutes*, *Proteobacteria*, *Actinobacteria*, and *Bacteroidetes (*43). The infant's gastrointestinal tract is initially an aerobic environment at birth, which promotes the development of facultative anaerobes or aerobes, such as *Firmicutes* (e.g., *Streptococcus*, *Enterococcus*, *Staphylococcus*) and *Proteobacteria* (e.g., *Klebsiella*, *Escherichia*, *Enterobacter*) (44). As oxygen levels in the intestine decrease, obligate anaerobes like *Actinobacteria* (e.g., *Bifidobacterium*) and *Bacteroidetes* (e.g., *Bacteroides*) become more abundant (45). The pharyngeal microbiota of infants was initially dominated by *Proteobacteria*, followed by *Firmicutes* becoming an absolute dominant phylum (46). Preterm infants often have lower microbial diversity, increased colonization of potentially pathogenic bacteria like *Proteobacteria*, and reduced levels of obligate anaerobes like *Bifidobacterium* and *Bacteroides (*47).

Through microbial composition analysis, our study showed that at the genus level, *Pseudomonas* and *Staphylococcus* dominate in both throat and stool samples. However, *Streptococcus* had a higher relative abundance in throat samples compared to stool samples (*p* = 0.003). At the phylum level, the dominant microbial communities in intestines and pharynges of preterm infants were similar: with the presence of *Proteobacteria*, *Firmicutes*, and *Bacteroidota*. This finding is consistent with the findings of Marsland et al. (48). The results of the study found that dominant species were present in many of the samples, with dominant bacteria present in 26 of the 28 pharyngeal samples. This is consistent with previous findings in the field (49). *Proteobacteria*, *Firmicutes*, and *Bacteroidota* make up the majority of human intestinal microbiota. Among them, *Proteobacteria*, which include facultative anaerobes, can be pathogenic and are associated with inflammatory bowel disease (IBD) (50), NEC (51), and obesity (52). *Firmicutes*, in higher abundance compared to *Bacteroidetes*, can extract energy more efficiently from diet thus potentially leading to obesity (53). *Bifidobacteria* can stimulate the production of fucosylated glycans by intestinal epithelial cells, promoting the colonization of beneficial microbiota and inhibiting the invasion of pathogens (54). They can also utilize fucosylated glycans to synthesize their cell walls, facilitating their growth and maintaining intestinal homeostasis (55). Beyond the age of seven, the ratio between *Firmicutes* and *Bacteroidetes* tends to remain relatively stable. An imbalance in this ratio may lead to metabolic syndromes such as obesity and diabetes. In line with the findings of Grier et al. (56), this study discovered that preterm infants predominantly harbor potentially pathogenic bacteria like *Firmicutes* and *Proteobacteria*, while beneficial bacteria like *Bifidobacterium* and *Lactobacillus* are less abundant. This suggests that preterm infants have a substantial presence of potentially pathogenic microbiota, which could predispose them to various diseases.

In newborns delivered vaginally, the early colonization of the intestinal microbiota is primarily influenced by maternal microbiota from the intestines, vagina, skin, and other sources. In contrast, infants born via cesarean section lack direct contact with their mother's microbiota during birth, potentially affecting the initial colonization of their intestinal microbiota (57). This delay in colonization might result in a delayed establishment of intestinal microbiota like *Bifidobacterium* and *Bacteroides* (58). Previous research has largely focused on the impact of delivery mode on the colonization of the intestinal microbiota, with little discussion on its influence on the pharyngeal microbiota. Our study found that in both the cesarean section and vaginal delivery groups, the dominant phyla in the pharyngeal microbiota were *Firmicutes*, *Proteobacteria*, *Bacteroidota*, and *Actinobacteriota*. There were no statistically significant differences in the composition at the phylum level in relative abundance or the alpha and beta diversity between the two delivery groups. However, at the genus level, the relative abundance of *Streptococcus* in the throat samples from the cesarean section group was higher than that in the vaginal delivery group (*p* = 0.01). *Streptococcus* is a potentially pathogenic genus that may pose a health risk, suggesting that babies born via cesarean section may be more susceptible to illness than those born vaginally. Bosch AATM et al. showed that mode of delivery affects the development of the early pharyngeal microbiota, especially the colonization of potentially protective commensal bacteria, and found that cesarean section increases the incidence of respiratory illnesses, and that cesarean section infants have delayed development of the pharyngeal microbiota in general, and reduced colonization of health-related commensal bacteria (e.g., Corynebacterium and Dolichoglossum) in particular, which may affect their respiratory health later in life (59). In this study, the intestinal microbiota was not detected in the vaginal delivery group, possibly due to the small sample size and the fact that the intestinal microbiota had not yet been colonized.

One of the strengths of this study is comparing intestinal and pharyngeal microbiota in preterm infants on the first day of life, as well as investigating the characteristics of pharyngeal microbiota in infants delivered by cesarean section or vaginally. Our results suggest a high overall degree of similarity in microbial composition in intestines and pharynges at birth. This indicates that the intestinal and pharyngeal microbiota showed similarity at birth and may be homologous, but further research is needed to determine the specific source. The most prominent strength of this study is the selection of late preterm infants as the study population. Late preterm infants have been excluded from many microbiological studies because they are usually considered full-term (i.e., normal infants) (8, 60), but they are at higher risk for health problems than full-term infants and cannot be considered “normal,” so this population was selected for the present study, which is expected to highlight the microbiological distribution of this group. Another advantage is that all pharyngeal samples were collected within 30 min of birth, and all stool samples were collected within the first day of life from the first meconium. An important consideration for a stool microbiome study is the time of sampling. Meconium is usually defined as the first stool passed within 48 h of birth (61). All stool samples in this study were collected within 24 h of the first meconium. The neonatal microbiome begins to diversify rapidly after birth, and it would be expected that a longer sampling time might result in samples containing bacteria acquired after birth. However, in an earlier study (62), it was shown that sampling times of 24 h after birth and shorter did not affect the bacterial load of the samples. Therefore, the results of the present study better reflect the characteristics of the distribution of the bacterial microbiota in preterm infants themselves.

Our study has some limitations as well. Firstly, our sample size is limited. Secondly, we had a limited observation period and hence did not capture the long-term effects of delivery mode on the pharyngeal microbiota. We were unable to observe changes in the microbiota over time. In future research, we plan to collect stool and throat swab samples in the days following birth to compare the changes in microbiota across different groups. Additionally, while it is widely believed that colonization of the infant microbiota begins in the first few days after birth, previous research on the microbiomes in the placenta and amniotic fluid suggests that the initial colonization of the infant microbiota may occur *in utero* (56). In future studies, we intend to collect amniotic fluid and placental tissue samples for microbial analysis to further explore the sources of human microbiota initial colonization. Previous researchers have studied the first meconium and used it as evidence of intrauterine fixation (61). The stool samples in this study were all first meconium collected within 24 h, but because of the lack of inclusion of information on amniotic fluid and placenta and the small sample size, it is only an overview and guide to intrauterine fixation, and it cannot be used as a complete evidence of intrauterine fixation. Geography is known to be an important factor in microbiome research. The subjects recruited for this study were all preterm infants born in Chengdu, so it cannot fully represent the distribution characteristics of the microbiota of preterm infants in all regions. A multicenter study will be considered in the future to have a more comprehensive understanding of the microbiota distribution characteristics of late preterm infants.

## Conclusions

In summary, the results of this study indicate that birth weight, gender, and delivery method do not have a significant impact on the microbial colonization of premature infants on the first day of life. Gestational age at birth may influence microbial colonization, but further validation is required with a larger sample size. Additionally, some newborns have evidence of microbial colonization in the intestine and pharynx at birth. Overall, the microbial diversity, composition, and relative abundance in the intestinal and pharyngeal microbiota of these infants show a high degree of similarity. However, due to the small sample size, further validation with a larger sample size is required to confirm this finding.

## Data Availability

The datasets presented in this study can be found in online repositories. The names of the repository/repositories and accession number(s) can be found in the article/Supplementary Material.
