# Supplementary material for: Comparison of intestinal and pharyngeal microbiota in preterm infants on the first day of life and the characteristics of pharyngeal microbiota in infants delivered by cesarean section or vaginally
Source: Front Pediatr. 2024 Oct 8;12:1411887. doi: 10.3389/fped.2024.1411887 (PMC11493734; doi:10.3389/fped.2024.1411887)
Supplement: Supplementary file 1 [file Datasheet1.zip › Data Sheet 1_v1/supplement/Laboratory procedures and bioinformatic data supplementation.docx]

Specifically, the primer sequences are not provided, the use of negative controls is not mentioned, the number of reads obtained per sample is not mentioned, number of reads in negative controls, the criteria used to determine which samples had a microbiota successfully detected is not clear and the use of rarefaction (or other standardisation technique, if used) prior to comparisons of diversity between samples is also not described. Particularly when working with low-biomass/low bacterial load samples, as in this study, it is crucial to have this information for the reader to be convinced the detected organisms are genuine microbiota and not the result of contamination.

Reply：

1. The primer sequences：F: CCTAYGGGRBGCASCAG; R: GGACTACNNGGGTATCTAAT;
2. Negative controls： There will be a negative control in the amplification session during the experiment of the amplification sub-project. The negative control is to replace the DNA sample in the PCR system with ddH2O, and other things remain unchanged. The negative control only exists in the amplification process and does not participate in the subsequent library construction, i.e., it will not be sequenced.

Reads obtained per sample: The number of readings for each sample is shown in the REPORT inside the Supplementary Materials.

Number of reads in negative controls: There will be no reads because the negative control will not be sequenced.

Which samples had a microbiota successfully: The amplification sub-project will also set up a negative control in the amplification session during the experiment, which is a negative control that replaces the DNA sample in the PCR system with ddH2O, while keeping the rest unchanged. The negative control only exists in the amplification process, and does not participate in the subsequent library construction, i.e., it will not be sequenced. The purpose of setting the negative control is to confirm that the measured data are the microbial community of the sample itself.

Comparisons of diversity：Data are homogenized before comparing sample diversity. Amplification sub-projects are first homogenized to the minimum amount of sequencing data for each sample before subsequent analysis of the information.
