# Supplementary material for: Comparison of intestinal and pharyngeal microbiota in preterm infants on the first day of life and the characteristics of pharyngeal microbiota in infants delivered by cesarean section or vaginally
Source: Front Pediatr. 2024 Oct 8;12:1411887. doi: 10.3389/fped.2024.1411887 (PMC11493734; doi:10.3389/fped.2024.1411887)
Supplement: Supplementary file 1 [file Datasheet1.zip › Data Sheet 1_v1/supplement/Novogene 16S Amplicon QIIME2 Analysis Report.docx]

Novogene 16S Amplicon QIIME2 Analysis Report

[16S.X101SC22102410-Z01-J002.report\report.html](肠道微生物文章/合同/16S.X101SC22102410-Z01-J002.report_20230120130810(1)/16S.X101SC22102410-Z01-J002.report/report.html)

1. Introduction

16SrRNA is a subunit of the RNA of the ribosome, and 16SrDNA is the gene that encodes this subunit. 16S rDNA consists of 10 Conserved Regions and 9 Hypervariable Regions, of which the Conserved Regions do not vary much among bacteria, and the Hypervariable Regions are genus or species-specific. There are some differences with different affinities. Therefore, 16S rDNA can be used as a characteristic nucleic acid sequence to reveal biological species, and it is considered to be the most suitable indicator for bacterial phylogeny and taxonomic identification. 16S rDNA Amplicon Sequencing usually selects certain or several variant regions, uses the conserved regions to design universal primers for PCR amplification, then analyzes and bacteriophage sequencing for the highly variable regions. The 16S rDNA Amplicon Sequencing has become an important tool to study the composition and structure of microbial communities in environmental samples^[1,2,3]^.

With the continuous development of high-throughput sequencing platforms, the upgraded NovaSeq sequencing platform can realize the PE250 strategy of double-end sequencing, which achieves similar read lengths as the MiSeq platform and greatly improves the throughput and sequencing quality compared with the MiSeq platform and becomes a new platform that is more suitable for 16S amplicon sequencing. The high throughput and depth of sequencing of NovaSeq PE250 are more favorable to identifying low abundance community species, thus improving the completeness of microbial community research, and will become the preferred strategy to study the diversity of microbial communities. NovaSeq PE250 will become the first choice for studying microbial community diversity because of its high sequencing throughput and depth, which will facilitate the identification of low-abundance community species and improve the completeness of microbial community studies.

A small fragment library is constructed according to the characteristics of the amplified region, and double-end sequencing is performed on the library based on the Illumina NovaSeq sequencing platform. The double-ended reads obtained by sequencing will be spliced, filtered, and reduced noise, and then the valid data obtained will be subjected to species annotation and abundance analysis, to reveal the species composition of the samples; and further analysis of α-diversity and β-diversity can not only excavate the differences in the structure of the communities among the samples, but also carry out personalized analysis and in-depth data mining according to the needs of the project.

1.1 Experimental process

In the process from DNA samples to final data acquisition, each step of sample testing, PCR, purification, library construction, and sequencing will have an impact on the quality and quantity of data, which in turn will directly affect the results of subsequent information analysis. To ensure the accuracy and reliability of the sequencing data from the source, NOVAX has strictly controlled each experimental step of sample testing, library construction, and sequencing, which fundamentally ensures the output of high-quality data.

- 1. Analyzing Processes

The raw data obtained from sequencing (Raw Data), there is a certain proportion of interference data (Dirty Data), to make the results of information analysis more accurate and reliable, firstly, the raw data are spliced and filtered to obtain valid data (Clean Data); then based on the valid data through DADA2 or deblur for noise reduction (the default use of DADA2) The final ASVs are obtained by splicing and filtering out sequences with abundance less than 5 ^[4]^. For the obtained ASVs ^[5]^, on the one hand, the representative sequences of each ASV are annotated with species to get the corresponding species information and species-based abundance distribution. At the same time, the ASVs are analyzed by abundance, Alpha diversity calculation, Venn diagram, and pentagram to get the information on species richness and evenness within the samples, the information of shared and endemic ASVs among different samples or subgroups, and so on. On the other hand, ASVs can be compared in multiple sequences phylogenetic trees can be constructed, and the differences in community structure among different samples or groupings can be explored through dimensionality reduction analyses such as PCoA, PCA, and NMDS, and the display of sample clustering trees. To further explore the differences in community structure among grouped samples, statistical analysis methods such as T-test, MetaStat and LEfSe were chosen to test the significance of differences in species composition and community structure of grouped samples. The annotation results of the amplicons can also be associated with the corresponding functional databases, and software such as PICRUSt2, BugBase, and Tax4fun can be selected to analyze the functional prediction of microbial communities in ecological samples.

2 Data processing

2.1 Data splicing

The raw data of each sample was first obtained by splitting according to the barcode and removing the barcode and primers, followed by splicing the R1 and R2 sequence data by FLASH software.

2.2 Data filtration

The spliced Tags were subjected to quality control to obtain Clean Tags. Then chimera filtering was performed to obtain valid data (Effective Tags) that could be used for subsequent analysis. The results of the statistics obtained from each step in the data processing process are shown in Table 1.

3 ASV analysis

3.1 Noise reduction and species annotation

The DADA2 method ^[6]^ is mainly used for noise reduction, which no longer clusters by similarity and only performs de-weighting (dereplication) or the equivalent of clustering by 100% similarity. Each de-emphasized sequence produced after noise reduction using DADA2 is called ASVs (Amplicon Sequence Variants) or feature sequences (corresponding to OTU representative sequences) and the table of abundance of these sequences in the sample is called the feature table (corresponding to the OTU table). The DADA2 method is more sensitive and specific than the traditional OTU method and can detect the real biological variants missed by OTU methods while outputting fewer false sequences ^[7]^. Meanwhile, ASVs instead of OTU improve the accuracy, comprehensiveness, and reproducibility of marker gene data analysis ^[8]^.

The QIIME2 classify-sklearn algorithm ^[9,10]^ was used to annotate species for each ASV using a pre-trained Naive Bayes classifier.

Based on the annotation results of ASVs and the characterization table of each sample, the species abundance tables at the level of kingdom, phylum, order, family, genus, and species were obtained, and these abundance tables with annotated information were the core content of the amplicon analysis. According to different experimental purposes, one or several key species can be screened from the species abundance tables at each taxonomic level (usually focusing on the phylum and genus levels), and combined with the results of species composition and difference analysis of different samples (groups), cluster analysis and other results for in-depth study

3.2 Relative species abundance

Based on the results of species annotation at different taxonomic levels, a bar chart of species relative abundance was generated to visualize the species composition and its proportion for each sample.

Based on the species annotation results, the top 10 species in terms of maximum abundance at each taxonomic level (Phylum, Class, Order, Family, Genus, Species) were selected for each sample or subgroup, and a bar chart of relative abundance was generated, so that we could visualize the species and their proportions of higher relative abundance at different taxonomic levels among the samples.

3.3 Species Abundance Clustering

Based on the species annotations and abundance information of all samples at the genus level, the top 35 genera in terms of abundance were selected and clustered at the species level according to their abundance information in each sample, which was plotted as a heat map to facilitate the discovery of the high and low levels of species aggregation content in each sample.

3.4 Venn/Petal Plot

According to the results of ASVs obtained from noise reduction and research needs, the ASVs that are common and unique among different samples (groups) are analyzed, and when the number of samples (groups) is less than or equal to 5, it is plotted as a Venn Graph, and when the number of samples (groups) is greater than 5, it will show a petal graph.

3.5 Species evolutionary tree at the genus level

In order to further investigate the phylogenetic relationships of species at the genus level, representative sequences of the top 100 genera were obtained by multiple sequence comparison.

4 Alpha polymorphism analysis

Alpha Diversity is used to analyze the diversity of microbial communities within a sample ^[11]^, and a single-sample diversity analysis (Alpha Diversity) can reflect the richness and diversity of microbial communities within a sample, including a series of statistically analyzed indices, species diversity curves, and species cumulative box plots to assess the species richness and diversity of microbial communities across samples of the Differences.

4.1 Alpha Diversity Indices

The Alpha Diversity analysis indices (observed_otus, Shannon, Simpson, chao1, goods_coverage, dominance, and pielou_e) for the different samples were counted and are shown in Table 2.

4.2 Species Diversity Curve

The Rarefaction Curve is a common curve that describes the diversity of samples within a group, It is constructed by randomly extracting a certain amount of sequencing data from a sample, counting their alpha diversity index values, and constructing the curve with the amount of sequencing data extracted versus the corresponding index value (cutoff=7576)

Species Accumulation Boxplot is an analysis that describes the increase in species diversity with increasing sample size. It is an effective tool for investigating the species composition of samples and predicting the abundance of species in samples and is widely used in biodiversity and community surveys to determine the adequacy of sample sizes and to estimate species richness. Richness) estimation in biodiversity and community surveys. Therefore, the cumulative box plot can not only determine whether the sample size is sufficient but also predict species richness if the sample size is sufficient (analyzed by default when the sample size is greater than 10).

4.3 Analysis of differences in the Alpha diversity index

In the analysis of differences between groups of Alpha diversity index, box plots can visualize the median, dispersion, maximum, minimum, and outliers of species diversity within a group. At the same time, whether the differences in species diversity between groups were significant was analyzed by T-test, Wilcoxon rank sum test, and Tukey test (T-test and Wilcox rank sum test when there were only 2 subgroups, and Tukey and Kruskal-Wallis rank sum test when the subgroups were larger than 2).

5 Beta polymorphism analysis

Beta Diversity is a comparative analysis of the microbial community composition of different samples. Firstly, based on the species annotation results of all samples and the abundance information of ASVs, the information of ASVs with the same classification was combined and processed to obtain the species abundance information table (Profiling Table). Meanwhile, the phylogenetic relationship between ASVs was utilized to further calculate the Unifrac Distance (Unweighted unifrac) ^[12,13]^. Unifrac Distance is a method of calculating inter-sample distances by using the evolutionary information between the microbial sequences in each sample, and for more than two samples, a distance matrix was obtained. Then, Weighted unifrac distance (Unweighted unifrac) was further constructed using the abundance information of ASVs ^[14]^. Finally, the Unifrac distance was analyzed by Beta Diversity Index intergroup difference analysis, multivariate statistical methods Principal Component Analysis (PCA, Principal Component Analysis), Principal Co-ordinates Analysis (PCoA, Principal Co-ordinates Analysis), and Non-Metric Multi-Dimensional Scaling (NMDS, Non- Metric Multi-Dimensional Scaling), etc. from which differences between different samples (groups) were found.

5.1 PCoA Analysis

Principal Co-ordinates Analysis (PCoA, Principal Co-ordinates Analysis) ^[15]^, is to extract the most dominant elements and structures from multidimensional data through a series of eigenvalues and eigenvectors sorting. We performed PCoA analysis based on Weighted unifrac distance and Unweighted unifrac distance and selected the combination of principal coordinates with the largest contribution rate for graphical presentation. If the samples are closer together, it means that the species composition is more similar in structure, so samples with high similarity in community structure tend to be clustered together, and samples with great differences in communities will be farther apart.

For PCoA, two forms of presentation are used, two-dimensional PCoA graphs are displayed with the first and second principal coordinates, while three-dimensional PCoA graphs are displayed with three principal coordinates in an interactive web page, and the coordinates can be flexibly adjusted.

5.2 PCA analysis

Principal Component Analysis (PCA, Principal Component Analysis) ^[16],^ is a method of downscaling multidimensional data based on the relative abundance distribution of ASV, to extract the most significant elements and structures in the data ^[17]^. By applying PCA analysis, the two coordinate axes that maximize the differences between samples can be extracted, so that the differences in multidimensional data can be reflected on a two-dimensional coordinate graph, thus revealing simple laws in the context of complex data. If the community compositions of the samples are more similar, the closer they are to the PCA plot

5.3 NMDS analysis

Non-Metric Multi-Dimensional Scaling (NMDS, Non-Metric Multi-Dimensional Scaling) ^[18]^ statistic is an ordination method suitable for ecological studies.NMDS is a nonlinear model based on Weighted_unifrac and Unweighted_unifrac distances to perform analysis based on the species information contained in the sample, reflected as points on a two-dimensional plane. It is designed to overcome the shortcomings of linear models (including PCA and PCoA) and better reflect the nonlinear structure of ecological data ^[19]^. NMDS analysis was applied to reflect the species information contained in the samples in the form of points on a multidimensional space according to the species information contained in the samples, while the degree of difference between different samples was reflected by the distance between points, and reflected the inter- and intra-group differences of the samples, etc.

6 Test of significance of differences in community structure between groups

Differences in community structure between groups were analyzed for significance by Weighted_unifrac and Unweighted_unifrac distances in Adonis and Anosim analysis methods.

6.1 Adonis analysis

ADONIS ^[20]^, also known as permutational MANOVA or nonparametric MANOVA, is a nonparametric multivariate analysis of variance method based on the Unifrac distance. This method analyzes the degree to which different subgroups of factors explain the differences in the samples and analyzes the statistical significance of the subgroups using the permutation test for significance ^[21,22,23,24]^.

6.2 Anosim analysis

Anosim analysis ^[25]^ is a nonparametric test based on the Unifrac distance to test whether the difference between groups is significantly greater than the difference within groups to determine whether the subgroups are meaningful.

7 Methodology: see the methodology section for details（Methods_QIIME2_EN pdf.）

8 Reference

[1] Caporaso, J. Gregory, et al. Global patterns of 16S rRNA diversity at a depth of millions of sequences per sample. Proceedings of the National Academy of Sciences 108.Supplement 1 (2011): 4516-4522.

[2] Youssef, Noha, et al. Comparison of species richness estimates obtained using nearly complete fragments and simulated pyrosequencing-generated fragments in 16S rRNA gene-based environmental surveys. Applied and environmental microbiology 75.16 (2009): 5227-5236.

[3] Hess, Matthias, et al. Metagenomic discovery of biomass-degrading genes and genomes from cow rumen. Science 331.6016 (2011): 463-467.

[4] Li Minjuan,Shao Dantong,Zhou Jiachen et al. Signatures within esophageal microbiota with progression of esophageal squamous cell carcinoma.[J] .Chin J Cancer Res, 2020, 32: 755-767.

[5] Callahan B J, McMurdie P J, Holmes S P. Exact sequence variants should replace operational taxonomic units in marker-gene data analysis[J]. The ISME journal, 2017, 11(12): 2639-2643

[6] Callahan, Benjamin J., Paul J. McMurdie, Michael J. Rosen, Andrew W. Han, Amy Jo A. Johnson, and Susan P. Holmes. DADA2: high-resolution sample inference from Illumina amplicon data.” Nature methods 13, no. 7 (2016): 581.

[7] Callahan B J, Wong J, Heiner C, et al. High-throughput amplicon sequencing of the full-length 16S rRNA gene with single-nucleotide resolution[J]. Nucleic acids research, 2019, 47(18): e103-e103.

[8] Amir A, McDonald D, Navas-Molina J A, et al. Deblur rapidly resolves single-nucleotide community sequence patterns[J]. MSystems, 2017, 2(2).

[9] Bokulich NA, Kaehler BD, Rideout JR, et al. Optimizing taxonomic classification of marker‐gene amplicon sequences with QIIME2’s q2‐feature‐classifier plugin. Microbiome. 2018a;6:90.

[10] Bolyen, E., Rideout, J.R., Dillon, M.R. et al. Reproducible, interactive, scalable and extensible microbiome data science using QIIME 2. Nat Biotechnol 37, 852–857 (2019).

[11] Li, Bing, et al. Characterization of tetracycline resistant bacterial community in saline activated sludge using batch stress incubation with high-throughput sequencing analysis. Water research 47.13 (2013): 4207-4216.

[12] Lozupone, Catherine, and Rob Knight. UniFrac: a new phylogenetic method for comparing microbial communities. Applied and environmental microbiology 71.12 (2005): 8228-8235.

[13] Lozupone, Catherine, et al. UniFrac: an effective distance metric for microbial community comparison. The ISME journal 5.2 (2011): 169.

[14] Lozupone, Catherine A., et al. Quantitative and qualitative β diversity measures lead to different insights into factors that structure microbial communities. Applied and environmental microbiology 73.5 (2007): 1576-1585.

[15] Minchin P R. An evaluation of the relative robustness of techniques for ecological ordination[J]. Vegetatio, 1987, 69(1/3):89-107.

[16] Jolliffe I T. Principal component analysis[J]. Journal of Marketing Research, 1986, 87(100):513.

[17] Avershina, Ekaterina, Trine Frisli, and Knut Rudi. De novo Semi-alignment of 16S rRNA Gene Sequences for Deep Phylogenetic Characterization of Next Generation Sequencing Data. Microbes and Environments 28.2 (2013): 211-216.

[18] J. B. Kruskal. Nonmetric multidimensional scaling: A numerical method[J]. Psychometrika, 1964, 29(2):115-129.

[19] Magali Noval Rivas, PhD, Oliver T. Burton, et al. A microbita signature associated with experimental food allergy promotes allergic senitization and anaphylaxis. The Journal of Allergy and Clinical Immunology.Volume 131, Issue 1, Pages 201-212, January 2013.

[20] Stat M, Pochon X, Franklin E C, et al. The distribution of the thermally tolerant symbiont lineage ( Symbiodinium, clade D) in corals from Hawaii: correlations with host and the history of ocean thermal stress[J]. Ecology & Evolution, 2013, 3(5):1317-1329.

[21] Anderson, M.J. 2001. A new method for non-parametric multivariate analysis of variance. Austral Ecology, 26: 32-46.

[22] McArdle, B.H. and M.J. Anderson. 2001. Fitting multivariate models to community data: A comment on distance-based redundancy analysis. Ecology, 82: 290-297.

[23] Warton, D.I., Wright, T.W., Wang, Y. 2012. Distance-based multivariate analyses confound location and dispersion effects. Methods in Ecology and Evolution, 3, 89-101.

[24] Zapala, M.A. and N.J. Schork. 2006. Multivariate regression analysis of distance matrices for testing associations between gene expression patterns and related variables. Proceedings of the National Academy of Sciences, USA, 103:19430-19435.

[25] M. G. Chapman, A. J. Underwood. Ecological patterns in multivariate assemblages: information and interpretation of negative values in ANOSIM tests[J]. Marine Ecology Progress, 1999, 180(3):257-265.

Table1.Statistical results obtained at each step of the data-processing process


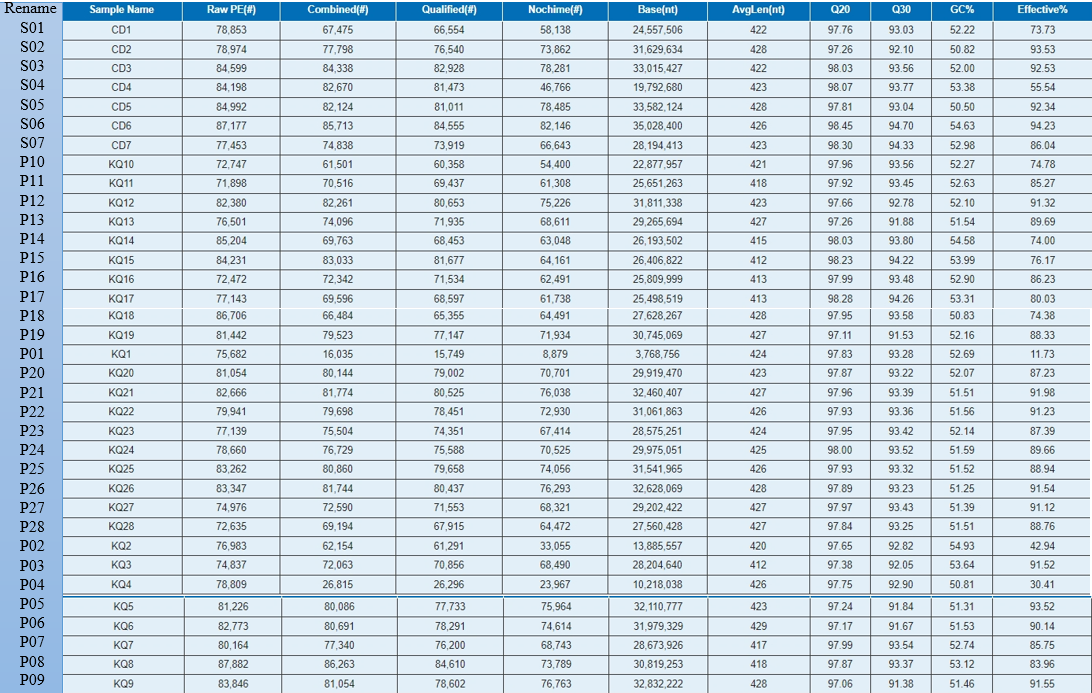


Description：Raw PE indicates the original downstream PE reads; Combined is the Tags sequence obtained by splicing; Qualified is the sequence after filtering low quality and short length of Raw Tags; Nochime is the final Tags sequence used for subsequent analysis after filtering chimeras, i.e., Effective Tags; Base is the number of bases of the final number of bases in Effective Tags; AvgLen is the average length of Effective Tags; Q20 and Q30 are the percentages of bases with base quality values greater than 20 (sequencing error rate less than 1%) and 30 (sequencing error rate less than 0.1%) in Effective Tags; and GC (%) indicates the number of GC bases in Effective Tags. GC bases in Tags; Effective(%) indicates the percentage of the number of Effective Tags to the number of Raw PEs.

Table 2. Alpha Diversity Index Statistics


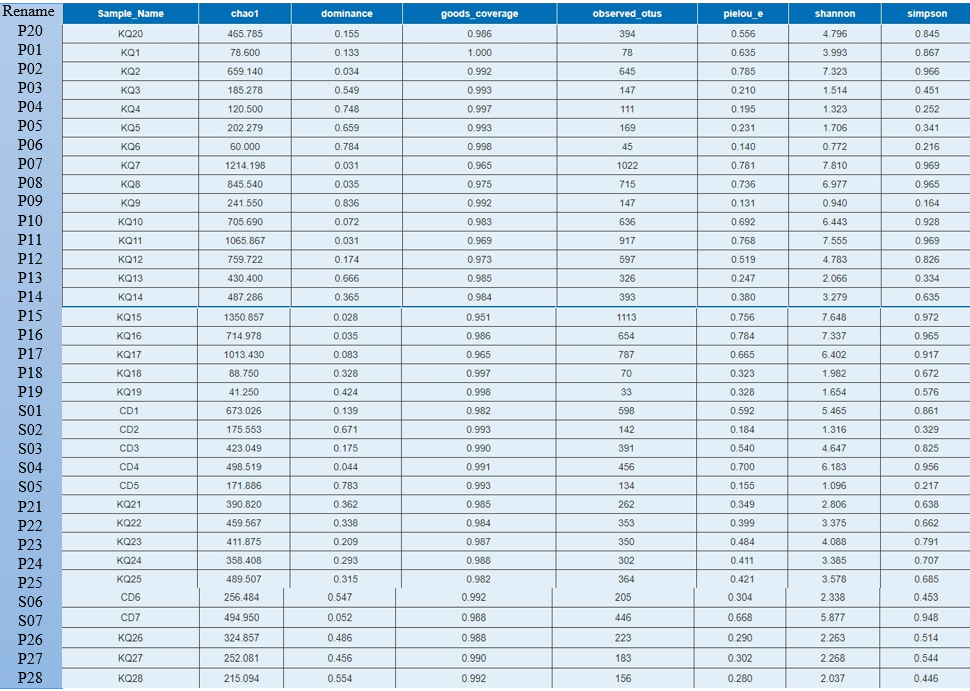


Description: chao1: estimate the total number of species contained in the community sample, the more low abundance species in the community, the larger the chao1 index; dominance: randomly take two sequences, the probability of coming from different species, the better the uniformity of the community species, the smaller the index; goods_coverage: coverage, the higher the coverage of the sequencing, the larger the index; observed_ otus: the number of visually observed species, the larger the index, the more observed species; pielou_e: evenness index, the more uniform the species, the larger the pielou_e; Shannon: the total number of taxa in the sample and its proportion. The higher the diversity of the community, the more uniform the distribution of species, the larger the Shannon index; Simpson: characterize the diversity and uniformity of species distribution within the community, the better the uniformity of species, the larger the Simpson index.
